# Genomics in animal breeding from the perspectives of matrices and molecules

**DOI:** 10.1186/s41065-023-00285-w

**Published:** 2023-05-06

**Authors:** Martin Johnsson

**Affiliations:** Department of Animal Breeding and Genetics, Swedish University of Agricultural Sciences, Box 7023, Uppsala, 75007 Sweden

**Keywords:** Genomics, Animal breeding, Genomic selection, Gene editing

## Abstract

**Background:**

This paper describes genomics from two perspectives that are in use in animal breeding and genetics: a statistical perspective concentrating on models for estimating breeding values, and a sequence perspective concentrating on the function of DNA molecules.

**Main body:**

This paper reviews the development of genomics in animal breeding and speculates on its future from these two perspectives. From the statistical perspective, genomic data are large sets of markers of ancestry; animal breeding makes use of them while remaining agnostic about their function. From the sequence perspective, genomic data are a source of causative variants; what animal breeding needs is to identify and make use of them.

**Conclusion:**

The statistical perspective, in the form of genomic selection, is the more applicable in contemporary breeding. Animal genomics researchers using from the sequence perspective are still working towards this the isolation of causative variants, equipped with new technologies but continuing a decades-long line of research.

## Background

Genomics, in the sense of genetic analyses using markers spaced out along the whole genome, has become a mainstream part of animal breeding. In March 2021, the dairy cattle evaluation in the US run by the Council on Dairy Cattle Breeding had accumulated five million genotyped animals [1]. These data are gathered for the purpose genomic selection, that is, evaluation of animals based on genome-wide DNA-testing, which was implemented in the US in 2007 (reviewed by [2]). Genomic selection builds on the practice of genetic evaluation by estimating a breeding value — a prediction of the trait values of the offspring that an animal will have — based on measurements on the animal itself and its relatives. Genomic selection adds molecular information in the form of genome-wide DNA markers to the evaluation.

Animal breeding before genomics was already immensely effective in changing the traits of farm animals. Take for example broiler chicken breeding. Zuidhof et al. [3] compared commercial broilers from 2005 (Ross 308 from Aviagen) with populations where breeding stopped in 1957 or 1978, kept in the same environment and fed the same feed. At eight weeks of age, the average body mass was 0.9 kg for the population with genetics from 1957, 1.8 for the population with genetics from 1978, and 4.2 kg for the population with genetics from 2005. The first SNP chip for chickens was developed in 2005 [4], and Aviagen started using genomic selection in 2012 [5] and thus, this difference is due to breeding that occurred before genomics. Genomics, however, made selection even more effective, either by increasing accuracy of selection or reducing generation interval, depending on the species. Potentially, it can also tell us about the molecular nature of the variants under selection and lead to new biotechnology applications for livestock.

The term “genomics” is derived from “genome”, which was coined by Hans Winkler in 1920 [6] and refers to one haploid set of chromosomes [7], or —with some degree of slippage in meaning — the complete DNA of a species. According to Thomas Roderick [8] the extension to “genomics” was conceived in 1986, as founders of the journal *Genomics* were trying to find a name for it. From the start, they regarded genomics as the name of a new field — “an activity, a new way to think about biology”.

There are (at least) two ways to think of genomics in animal breeding: two perspectives on genomics that will, throughout this paper, be called the *statistical* and the *sequence* perspectives:

1. We may think of the genome as a big table of numbers, where each row is an individual and each column a genetic variant, and the numbers are ancestry indicators. These matrices lend themselves to statistical calculations such as estimation of genomic breeding values. This is the view from the statistical perspective.
2. Alternatively, we may think of the genome as a long string of A, C, G and T. They lend themselves to molecular biology operations like predicting the amino acid substitution from a base pair substitution, or identifying patterns of interest. This is the view from the sequence perspective.

The perspectives roughly map to two concepts of a so-called gene [9]: The statistical perspective relates to the instrumental gene, a calculating device used by classical geneticists to understand inheritance patterns. The instrumental gene is a particle of inheritance, observed indirectly through crosses and comparisons of traits between relatives. For an example, the textbook of classical genetics by Sturtevant and Beadle [10] is full of crossing schemes of fruit flies that allow modes of inheritance to be investigated. In the introduction, the authors describe their view of genetics as a science. They call it “a mathematically formulated subject that is logically complete and self-contained”, without the necessity of a physical or chemical account of how inheritance works. On the other hand, the molecular perspective aligns closer with the nominal gene concept, where a gene is a DNA sequence that has a name and (potentially) a function. As an example, we can look at a genome browser such as Ensembl [11], which shows a genome as a series of track, with colourful boxes denoting genes, regulatory DNA sequences, and other associated information.

To be clear, I am not suggesting that individual geneticists are so limited in their thinking as to use only one of these perspectives. Any one researcher probably has these and several other mental models of the genome for different tasks. In practice, geneticists seem to routinely switch between different perspectives and conceptions of central terms like “genome”, “gene” and “locus”, without much friction. Certainly, ambiguity may lead to “complexity and confusion” [12], but I would argue that the imprecision is also sometimes productive, as it avoids unnecessary debates about which of these concepts are “right”, when the real answer is that all of them are working models and all are useful in different contexts.

The two perspectives lead to different views about the importance of identifying sequence variants that cause trait differences between individuals (“causative variants”, for short). From the statistical perspective, genomic data are large sets of markers of ancestry; we can make use of them while remaining agnostic about their function. From the sequence perspective, genomic data are a source of causative variants; we need to identify and make use of them. To realise the future potential of the sequence perspective, geneticists need to identify causative variants, while the statistical perspective has been successful, precisely by ignoring causative variants. The power of markers [13] is what Sturtevant & Beadle described: The point is to make use of statistical regularities without getting bogged down in mechanistic detail. Conversely, the potential of the molecular perspective is in understanding mechanisms and learning to manipulate them in ways that would not be possible by traditional selection and crossing. Mostly, this potential of the sequence perspective has not been realised, but the search for molecular knowledge has made possible tools that underpin applications of the statistical perspective, especially genomic selection.

## Main text

### Tools of the statistical perspective

Genomic selection is the crowning achievement of the statistical perspective on genomics in animal breeding, building on a long line of research of mapping phenotypes to genotypes. Genetic mapping — the family of methods used for localising variants that affect traits, roughly at first — goes back to the early history of classical genetics. Once geneticists had discovered that genes were arranged linearly on chromosomes, they could build maps of where causative variants underlying visible phenotypes were located relative to each other, the first map being published by Sturtevant [14]. This map building activity, based on crossing and detecting recombinant individuals, is called linkage mapping. The extension to complex traits with many causative variants of small effects is traditionally called “quantitative trait locus mapping” [15]. The extension to large population samples of more distantly related individuals is called “genome-wide association” [16], and has become the dominant form of genetic mapping. Arguably, genetic mapping can be viewed both from the statistical and sequence perspectives. On one hand, these methods involve statistical genetical methods that are very similar to those used in genomic prediction, and involve representing genomic data statistically. On the other hand, the end goal is usually to identify causative variants.

Out of genetic mapping of traits relevant to breeding comes marker-assisted selection, an earlier paradigm for incorporating molecular information in breeding. In a way, marker-assisted selection is the most intuitive way to imagine molecular breeding: Imagine that we have identified some genetic variants that either cause a trait of interest, or are strongly associated with it. Then, we can genotype our selection candidates for the variant of interest, and incorporate those genotypes into selection decisions. For example, if we know about a strongly deleterious variant, we can exclude candidates that carry it. The proposition of a genetic test is especially attractive when the trait is otherwise hard to phenotype. This was precisely the situation with several large-effect deleterious alleles in pigs and cattle, where marker-assisted selection was successfully implemented against the problematic alleles: malignant hyperthermia and the RN gene in pigs (reviewed by [13, 17]) and BLAD in cattle [18]. DNA tests for such large-effect damaging variants are now routinely included in many genomic breeding programs (e.g., [19, 20]).

At some point during the late 1990 to early 2000s, animal breeding researchers shifted their thinking from marker-assisted selection to genomic selection, from thinking about mapping causative variants to treating the whole genome together. Arguably, the key paper, and the most cited, is the one by Meuwissen, Hayes and Goddard [21]. It presents the full case for genomic selection, including simulations and a few alternative estimation methods (leading to the so-called Bayesian alphabet family of methods). However, genomic selection did not appear fully formed at once. Other genomic selection precursor papers from the era include:

- The 1990 paper by Lande & Thompson [22] that contains the key idea of covering the genome with markers and selecting on a total score based on all the markers.
- The 1997 paper by Nejati-Javaremi, Smith & Gibson [23], the key idea of which is to create a relationship matrix based on variants that affect a trait, creating estimated breeding values based on what they call “total allelic relationship”.
- The 1998 paper by Haley & Visscher [24] which uses the term “genomic selection” and clearly expresses the concept, including the interpretation of genetic markers as realised relatedness.

Exactly when and by whom (in conversation or in parallel) the shift happened is a topic of its own. It seems to have been a gradual process. Still, Meuwissen, Hayes and Goddard (2001) is a landmark in that it provided a full recipe for genomic selection, and ran the proof of concept *in silico*. Genomic selection worked well enough in theory that is provided the inspiration for creating the tools and the practical initiatives to make it reality.

We can think of genomic prediction it as refining the estimate of how closely related animals are to each other by observing how much DNA the animals share, as opposed to the average relatedness that can be predicted from a pedigree. Alternatively, we can think of it as simultaneously estimating the contribution of every part of the genome (that is, every marker we genotype), and adding them up to a genomic estimate for that animal (see [25] for a review of the statistical approaches used in animal breeding). Either way, the key insight in genomic selection is that one can accurately predict breeding values in the absence of information about the function of particular variants by combining all markers in one statistical model. As Lowe & Bruce point out [13], this black-boxing of genetic mechanisms is characteristic of the quantitative genetics tradition, here expressed by one of the pioneering applied quantitative geneticists, Lush [26]:

*It is rarely possible to identify the pertinent genes in a Mendelian way or to map the chromosomal position of any of them. Fortunately this inability to identify and describe the genes individually is almost no handicap to the breeder of economic plants or animals. What he would actually do if he knew the details about all the genes which affect a quantitative character in that population differs little from what he will do if he merely knows how heritable it is and whether much of the hereditary variance comes from dominance or overdominance, and from epistatic interactions between the genes.*

Lowe & Bruce argue that this attitude is key to the success of genomic selection: this strategy is the outcome of an alignment, but not a full integration of quantitative and molecular genetics, which allowed quantitative genetics to make use of molecular methods to generate ever denser marker maps, while sticking with the tradition of abstraction [13].

The effects of genomics have been dramatic. Genomic prediction allows selection to proceed more quickly, or more accurately, depending on the biology of the species and the design of the breeding program. In cattle, increased selection accuracy for young bulls without daughter records allow shorter generation times [2, 27, 28], and genotyping of heifers much improves selection accuracy of cows relative to pedigree-based evaluation [29]. In pigs, genomics have increased accuracy of selection in several traits by 50% [17]. In poultry, accuracy has also increased; a review of genomic selection in poultry gives accuracy increases ranging from 20% to over 50% in layers and broilers [5].

There are further statistical genetics tools, agnostic of marker function, that can be enriched by genomics. Optimal contributions selection (reviewed by [30]) is a family of methods to balance the genetic improvement and inbreeding or loss of diversity of a population. These methods work by finding less related individuals to pair, that still give a high expected genetic gain in the offspring. Like in genomic selection, pedigree relatedness can be substituted with genomic relatedness. Since genomic selection in practice tends to accelerate inbreeding, there may be greater need for optimal contributions selection in genomic breeding. Specifically, genomic selection can in principle differentiate between individuals that are identically related in terms of pedigree, and thus lead to less correlation between families, and a lower inbreeding rate, all else equal [31]. In practice, all else is not equal, because genomics leads to redesigns of breeding programs, which may in itself increase or decrease the inbreeding rate. In breeding programs where genomic selection helped reduce generation time, a low inbreeding rate per generation may translate to accelerating inbreeding per year. There are examples of both accelerated [32] and reduced inbreeding rates after genomic selection [33].

Furthermore, population genetic methods can find the similarity between populations and individuals, and classify individuals based on breed composition, geographic origin or assign offspring to parents. For example, DNA testing to confirm pedigree in cattle started with blood groups, moved on to genetic markers, and now use the genome-wide SNP chips that are used for genomic selection [34]. Genomics allows plentiful markers distributed throughout the genome, and so, methods can be more precise in pinpointing ancestry [35], and reconstruct pedigree information that is missing [36].

### Tools of the sequence perspective

From the sequence perspective, the development of genomics in animal breeding can be seen as ongoing effort to build the tools for causative variant identification. In the process, it also gave rise to the enabling technology for genomic selection. This development includes reference genomes for farm animals, dense marker panels and affordable methods to type them (SNP chips, reduced representation sequencing), genome annotation and maps that localise causative variants in the genome (linkage mapping and genome-wide association).

The chicken genome sequence was published in 2004 [37], cattle in 2009 [38], and pig in 2012 [39]. The choice of any one publication and year as a milestone in a genome sequencing project is somewhat arbitrary, because the sequences reported in these papers were neither the first nor the last drafts. Genome assembly is an iterative process that combines different kinds of data, computational models, and human judgement to represent a genome. For a historical account of the diverse data and ways of reasoning used in the pig genome project, see Lowe [40]. Lowe points out that a genome project was not just about sequencing in the narrow sense of putting DNA base pairs in order, but “thick” sequencing, which also includes the creation of tools, annotation with additional data, and dissemination to a research community that makes reference genomes useful. Consequently, the development of farm animal reference sequences is still ongoing, with the pig, cattle and chicken genomes being updated [41, 42] and followed by sheep, goat, ducks, turkeys and many other. There are now multiple high-quality genome assemblies, e.g. in cattle [43, 44]. Inevitably, more are coming, as genome assembly becomes more affordable and streamlined.

The next layer atop the reference genome is annotation, here understood as any information that has a genomic coordinate, localising it in the genome. As Szymanski et al. [45] point out in a study of the yeast genome, one of the functions of a reference genome as a digital model of the genome is to allow researchers to organise and connect different sources of data. Researchers can put their data on the same coordinate system and create a coherent picture. In the yeast community, that coherence-building used to be achieved by sharing strains and standard protocols, before the reference genome. For logistical reasons, germplasm sharing is harder in farm animal genetics. But now, genome annotation is available in genome browsers such as the NCBI Genome Data Viewer and Ensembl, which contain comparative information [46], the location of genes, and non-genic elements of importance such as open chromatin (as it is becoming available). Projects like Functional Annotation of Animal Genomes [47] are producing detailed maps of gene-regulatory regions in farm animal genomes, with the express purpose that researchers are going to be able integrate their openly available data into their projects. Such functional genomic data might be useful both for annotating genetic variants as a part of fine-mapping and nominating potential causative variants, in genomic prediction with sequence data, and in molecular biology studies of gene-regulatory networks.

The key technology, however, enabling genomics in farm animals is affordable high throughput genotyping, in the form of SNP chip technology that allows the testing of thousands of single nucleotide variants (SNPs) at the same time. SNP chips are, generally, surfaces with known pieces of DNA them. The array captures fragments of DNA close to the markers we want to type, and a DNA polymerase enzyme that incorporates labelled nucleotides gives a fluorescence signal, where the relative signal intensity of the alleles will tell us the genotype [48]. A clustering algorithm will help turn the intensity values into genotypes — the numeric coding needed for all the statistical genomic methods.

Looking at the original three farm animal genome papers, they all mentioned genetic improvement of livestock, but in oblique terms. It is as if they either did not know precisely how a reference genome would improve breeding in these animals, or that the way forward now that the reference genome was in place was too obvious to even to mention:

- *The chicken genome sequence promotes both the development of more refined polymorphic maps (see the accompanying paper* [49]*) and the framework for discovering the functional polymorphisms underlying interesting quantitative traits, thus fully exploiting the genetic potential of the chicken.* [37]
- *The cattle genome and associated resources will facilitate the identification of novel functions and regulatory systems of general importance in mammals and may provide an enabling tool for genetic improvement within the beef and dairy industries.* [38]
- *The pig genome sequence provides an important resource for further improvements of this important livestock species, and our identification of many putative disease-causing variants extends the potential of the pig as a biomedical model.* [39]

However, when the first SNP chips were being published, the design of the SNP chips were explicitly motivated with the ability to perform genomic selection, in addition to the ability to improve genetic mapping:

- *The aim of this study was to develop and characterize a high-density, genome-wide SNP assay for cattle with the power to detect genomic segments harboring inter-individual DNA sequence variation affecting phenotypic traits and for application to GWS, in which an animal’s genetic merit is estimated solely from its multilocus genotype.* [50]
- *The most efficient way to genotype large numbers of SNPs is to design a high-density assay that includes tens of thousands of SNPs distributed throughout the genome. These SNP “chips” are a valuable resource for genetic studies in livestock species, such as genomic selection, detection of [quantitative trait loci] or diversity studies.* [51]
- *In livestock species like the chicken, high throughput single nucleotide polymorphism (SNP) genotyping assays are increasingly being used for whole genome association studies and as a tool in breeding (referred to as genomic selection).* [52]

These genomic tools — reference genomes, genome annotation, large-scale genotyping — build towards detecting causative variants that affect traits by allowing bigger and more marker-dense genome-wide association studies for localising causative variants, and the ability to look under the loci detected to find the underlying genes and important sequence elements, such as gene-regulatory sequences. It is striking to read the attitudes in commentaries on genomics in animal breeding from the early days of genomics. Here is Bulfield [53] in 2000 describing the isolation of causative variants:

*Farm animal genomics is developing in four phases. (1) Constructing maps of highly informative markers and genes. (2) Using these maps to scan broadly across genomes of resource populations, segregating for commercially important traits, to locate quantitative trait loci (QTL) into 20–40 cM chromosomal segments. (3) Identifying the trait gene(s) themselves, within these regions. (4) Bridging the ‘phenotype gap’ between the gene(s) and the ultimate trait.*

What implications would this have for animal breeding? Bulfield continues:

*In animal breeding, a combination of genome analysis and cell culture-based transgenesis would permit a more controlled approach to animal breeding, especially for currently intractable traits such as fertility and disease resistance. In addition, cloning from adult cells (as with Dolly) would permit the replication of (for example) a proven high-yielding and productive dairy cow.*

On the same theme, Goddard [54] wrote in 2003:

*I believe animal breeding in the post-genomic era will be dramatically different to what it is today. There will be a massive research effort to discover the function of genes including the effect of DNA polymorphisms on phenotype. Breeding programmes will utilize a large number of DNA-based tests for specific genes combined with new reproductive techniques and transgenes to increase the rate of genetic improvement and to produce for, or allocate animals to, the product line to which they are best suited. However, this stage will not be reached for some years by which time many of the early investors will have given up, disappointed with the early benefits.*

In retrospect, Bulfield was clearly too optimistic; Goddard’s more tempered optimism might still be right depending on how long time counts as “some years”. Also, the technologies listed by Bulfield [53] — linkage maps of 20 to 40 cM resolution, microsatellite and amplified fragment length markers, back-crosses and expressed sequence tag libraries — sound antique to students of animal breeding educated today. The low number of markers (e.g., 40 cM resolution would mean about 150 markers to cover the cattle genome), made sense for genetic mapping based on linkage within families, which was the state of the art at the time. The tools of the sequence perspective have moved far during 20 years, but the underlying problems of causative variant identification remain the same.

That is, despite the increasing development of molecular tools, statistical methods, and increasing dataset sizes, there are few known causative variants for economically important traits (see tables in [55]). None of them have yet led to transgenic animals that are used in farming. Why have we not found the causative variants? There are at least three problems:

1. It turns out that most traits of interest are massively polygenic. That is, they are affected by thousands of genetic variants, most of individually small effects. This has been a staple assumption of quantitative genetics since the early 20th century, and was further cemented by the failure of linkage mapping to explain large chunks of inheritance, and now there are methods (based on genomic selection models) to estimate polygenicity from data. The estimated number of variants for complex traits in humans are in the range of tens of thousands of causative variants [56, 57].
2. Quantitative traits may have complex genetic architectures in other ways than polygenicity; they may be affected by rare variants whose effects are hard to estimate, and variants that act in non-additive ways (dominance or epistasis). This is less important for selection, as the response to selection depends on the additive genetic variance, and even non-additive effects at the variant level can result in substantial additive genetic variance [58, 59]. However, when we go on to identify causative variants, it may matter, for example, if the apparently additive outcome depends on pairwise interactions between variants that are located close together.
3. Even when an association has been isolated (and there are thousands of them [60]), fine-mapping an association signal down to the causative variant or even gene is hard, because there are many variants, and they correlate (geneticists call this correlation, abstrusely, “linkage disequilibrium”), and interpreting them and testing their effects are hard work.

The Goddard [54] quote is particularly apt, because while the post-genomic future he envisaged, based on the sequence perspective, has not happened, at about the same time as that paper was published, he was involved in developing genomic selection, the statistical genomics future that happened instead.

### Statistical futures

What is the future of genomic breeding? From the statistical perspective, the immediate future seems to hold even more genomic selection — on more data, with new traits, spread to new species and breeding programs, and possibly enhanced with functional genomic data.

As data accumulate on more and more animals, larger datasets cause computational difficulties. Methods such as APY (the “algorithm of proven and young”), which splits a genomic selection dataset into a “core” group of animals and a “peripheral” group of animals and performs the most intense computations only on the core subset, allow one to use large numbers of genotyped animals and still be able to compute estimated breeding values in reasonable times [61]. There is a whole strand of genomics research in animal breeding that works on improving the way genomic selection models are used in practice, how to fit the models efficiently, how to re-fit them when new data arrives, and how to estimate their accuracy (see review by [62]).

Another ongoing strand of research is extending genomic selection to more complicated genetic scenarios like crossbred animals or generalisation between different populations. Standard genomic selection models work best for prediction within a single population. Thus, if crossbred animals are used for breeding, as is common for example in beef cattle, one would like to have genomic estimated breeding values for them. Even when the crossbred animals might not be used in breeding themselves, such as in pig or poultry breeding, there are traits that can only be measured on crossbred individuals and that information needs to be propagated back to the purebred nucleus animals. Similarly, small breeds might struggle to gather enough data, and the ability to borrow information from larger breeds is attractive.

However, genetic distance between animals quickly reduces the accuracy of genomic selection, complicating across-breed and multi-breed genomic prediction (see review by [63]). First, comparing distantly related breeds, the marker—trait associations in each breed could be very different, both because the breeds might carry different causative alleles and because the correlations (linkage disequilibrium) between causal variants and markers might be different. Second, non-additive genetic effects, which to a first approximation can be discounted as a nuisance factor within a population, can make a substantial difference as genetic differences accumulate. To accurately predict the outcome, a full model would have to consider both dominance and the genotypes at multiple interacting loci. However, without identifying the interactions and non-linearities, the correlation between marker effect estimates can be shown to decline with genetic differentiation [64].

Another avenue of development is to find a place for machine learning methods in genomics of animal breeding. Machine learning methods have been used in functional genomics to predict variant effects (reviewed by [65]), and in animal breeding applications for developing new phenotypes [66, 67], but so far have not been widely used in genomic selection. This is not for lack of trying; early work included attempts at using kernel methods [68, 69], tree regression [70] and neural networks [71], and later efforts have been made with deep learning [72, 73]. However, unless we count linear mixed models as a machine learning application, these have not made much impact on applied genomic selection. Probably, this is because non-additive effects have hitherto not played a big role in selection, and these methods only outperform linear mixed models when predicting non-additive effects. This may change if genomic selection is extended to systems where non-additive effects are more important, and one has to design matings to produce offspring that deviate from the parent average in the right direction [74], or for applications where predicting individual phenotype rather than breeding value is the goal.

Finally, there is a strand of research that aims to improve genomic selection by adding more genomic information. For biological reasons, some variants are expected to contribute more — variants close to known associations from genome-wide association studies, variants predicted by bioinformatic means to be functional, variants associated with gene expression variation, variants located in open chromatin in a relevant tissue, and so on. Various statistical extensions to the genomic selection models allow groups of variants to be treated separately [75, 76] and given different emphasis depending on their predicted function. Such methods would be important for performing genomic selection with whole-genome sequence data, that include millions rather than tens of thousands of variants. It seems clear that there is potential. A series of studies using gene expression quantitative trait locus data in combination with chromatin and evolutionary conservation suggest that one might be able to prioritise variants that are more likely to explain quantitative trait variation [77, 78]. However, empirical results on whole-genome sequence data in genomic prediction [79–82] are inconsistent between methods, populations and traits about whether adding genomic information brings any benefit, or even degrades accuracy. Even in simulations where the causative variants are known [83], the increase in accuracy from including true causative variants is not great, unless the true effect sizes of the variants are known. Therefore, the potential gain from enhancing genomic selection is probably much less than from the improvement that came from starting genomic selection over traditional evaluation.

The statistical perspective also holds the opposite possibility for a turn away from the genome. Instead of pursuing more genomic data to possibly improve genomic prediction, one could invest in improving measurement technology or modelling to improve the measurement of traits. Because the task, from the statistical perspective, is not to understand the genome but to get a good enough estimate of ancestry, it might be that the best choice is to settle for a relatively crude genotyping strategy (like a medium density SNP chip) and instead focus on gathering more records on high-value but hard-to-measure traits [84].

### Sequence futures

As we saw above, around the turn of the century there was optimism about identifying causative variants and exploiting them in animal breeding, which turned out to be mostly premature. Marker-assisted selection was successfully used on large-effect variants such as genetic defects, but less successful for quantitative traits. There are thousands of quantitative trait loci and genome-wide association hits published for economically relevant quantitative traits in farm animals, but only a handful that have been fine-mapped down to a causative variant [85]. However, molecular genetic techniques have moved rapidly over the last 20 years, not just adding new assays for gene-regulatory activity, but scaling them to the whole genome. With these new tools at hand, researchers are again optimistic that causative variants can be identified and exploited.

Several papers outline a vision of a future for the sequence perspective in animal [86, 87] and plant breeding [88], using genome editing methods such as CRISPR/Cas9 to supplement classical breeding with causative variants of known function. They call future, causative-variant enabled breeding “Livestock 2.0” and “Breeding 4.0”. Beside the version number conflict the visions have a similar overall shape: the future of breeding lies in identifying genetic causative variants through large genomic datasets, and then introducing them into breeding individuals through gene editing. Clark et al. [86] also describe identifying functional variants and editing them as “a route to application” for functional genomic data in farm animals.

The first application along this route of gene editing would be the ongoing attempts at editing of monogenic high-value traits, such as hornlessness caused by *polled* alleles in cattle [89], or porcine reproductive and respiratory syndrome virus resistance in pigs conveyed by edits to the *CD163* gene [90]. In the case of pigs, the causative variant does not occur naturally, and was designed based on molecular knowledge about the virus’ mode of infection. The hornless variant (“polled”) was identified by genome-wide association [91]. Conceptually, these proposed applications are somewhat different than the applications that have been proposed for transgenic animals before. Transgenic farm animals, such as the defunct “Enviropig” project [92] or the AquaAdvantage salmon [93], would have DNA introduced from different species, and can be thought of as examples of a genetic engineering approach. These modern proposals typically use less dramatic changes, alleles that exist in nature, or could relatively easily happen by natural mutation (e.g., partial deletion of a gene in the *CD163* example, or producing a duplication similar to a naturally occurring duplication in the *polled* case).

Gene editing is like marker-assisted selection in the sense that the variants to be edited need to have large enough effects to be worthwhile, and editing must be more effective than conventional alternatives. Both resistance to porcine reproductive and respiratory syndrome and polledness are potentially traits of great value and connected to animal welfare. Outbreaks of porcine reproductive and respiratory syndrome has devastating consequences for pig health and farm profitability, and simulations suggest that gene editing in combination with partially protective vaccines could eliminate the disease [94]. Hornless cows are highly desirable by farmers and dehorning is a welfare issue. As for conventional alternative strategies, natural knockouts of the *CD163* gene in pigs appear to be exceedingly rare [95]. *Polled* alleles, however, occur in many breeds, including dairy breeds conceived as targets of editing, and marker-assisted selection is already in use in breeding programs to promote it, as polled status can be predicted from SNP chips used for genomic selection. Simulation studies suggest that an editing-based strategy for promoting *polled* can have better consequences in terms of genetic gain and inbreeding than marker-assisted selection [96–98], but it remains to be seen whether the technological hurdles, regulations, acceptability and ethical issues will be resolved in time for *polled* gene editing to be successful.

However, going beyond monogenic traits to complex traits, the lack of other routes to application other than gene editing becomes a problem. If editing or marker-assisted selection are the only applications for knowledge of causative variants, and neither is likely to work well for complex traits, this limits the applied potential of the sequence perspective. Molecular insights about traits in farm animals are scientifically interesting, but currently have little other applied value. This is often not very clear from reading genomic studies, that often promise improvements to animal breeding without spelling out how they will come about. Allow me a personal and somewhat embarrassing example: In the introduction to my PhD thesis, which was defended in 2015, I wrote about the quantitative trait loci that I had identified, and speculated about what would be needed for them to be used in actual breeding. This discussion was completely misguided. It raised true concerns, such as whether the association would replicate in a different population, whether the underlying variant between shared associations in different populations are the same, and so on, but it missed the mark, because I was not aware that marker-assisted selection for quantitative traits was essentially dead at this point. The quantitative trait locus paradigm that I was operating within was dead and buried in animal breeding, and the first commercial genomic selection of poultry was already happening [5].

Most traits of economic relevance to animal breeding are affected by many variants of small effects. This polygenicity means that in order to know what sequences to edit and what to put instead one needs to solve the fine-mapping problem, to find ways to reliably identify causative variants, even if they are of moderate effect size. The situation is more challenging than with marker-assisted selection, where it may be enough to detect a variant in close linkage disequilibrium with the genuine causative variant. It is still an open question when and how we will get detailed enough knowledge of the genomic basis of complex traits to do this. It would require a workflow to identify causative variants reliably enough to edit them, in a very short time compared to current methods where thorough characterization of a causative variant takes years.

Furthermore, pleiotropy and non-additive effects might affect predictability of the outcomes of editing. Because the size of the genome and its repertoire of genes is limited, genes and pathways are recycled in a context-dependent manner for many biological functions. This suggests that many genetic variants will affect multiple traits, likely mediated by gene-regulatory relationships. This postulate of “universal pleiotropy” goes back to early quantitative genetics [99] and forms part of the more recent “omnigenic model” of complex traits [100]. This suggests that any use of gene editing needs to be vigilant against side-effects and consider the whole breeding goal in a balanced way, as argued by [101]. In the presence of non-additive effects, the statistical effect of an allele substitution depends on the frequency of the interaction partners. This means that the net effect of a gene edit might change as the population changes, as argued by [101, 102]. However, one might argue that we already take genomic selection decisions, and thus shift the allele frequency of regions associated with large marker effects, on the basis of estimates that average over potential interactions and are liable to change over time.

The next problem to overcome is how to introduce many edits into a breeding program. The challenge has two parts: First, multiplex gene editing technically challenging on its own, given that the success rate of a biallelic homology-directed repair editing event with CRISPR/Cas9 is low. Even if it could be increase to double digits, the success rate for multilocus edits would scale poorly. Second, integrating gene editing into animal breeding programs would involve performing gene editing at the scale of many animals. Jenko et al. [103] suggested a strategy of promotion of alleles by gene editing, where the chosen sires of a breeding program would be edited to be homozygous for causative variants that they did not already carry. They assumed that causative variants were known and that sires could be selected before they were edited. This would require new reproductive technology integrated with genomic selection. Such in vitro breeding strategies have been proposed several times [24, 104, 105] as extensions of the already advanced reproductive technologies used in particular in cattle breeding. For example, if an embryo transfer is already in use to breed sires for a cattle breeding program, it might be possible in the future to use to introduce gene editing machinery into the embryo, then biopsy a small amount of DNA to both verify the integrity of the edits and perform genomic selection. It remains to be seen, if this strategy becomes technologically feasible, what numbers of edited embryos and what levels of failure of editing would be acceptable. The failure rate of gene editing technologies are currently high, and that may lead to high costs and loss of selection response [96].

Johnsson et al. proposed removal of deleterious alleles [106], reasoning that damaging variants might be easier to identify from sequence data than causative variants for quantitative traits, and that recessive deleterious alleles may be common in farm animal populations due to ineffective natural selection and the large impact of genetic drift. While that assumption may be true, there is currently no workflow for large-scale identification of deleterious variants in place, and when such variants are detected, marker-assisted selection is more attractive than gene editing.

In summary, the sequence perspective faces challenges, not just within genomics (the fine mapping problem) but also within reproductive technology and breeding program design (the problem of multiplex editing). Gene editing of very large-effect variants is somewhat akin to marker-assisted selection, where there are reliable workflows for causative variant identification, and individual effects may be dramatic enough to justify editing. However, gene editing of causative variants for complex traits appears to fraught with problems to be possible within the foreseeable future. Perhaps finding a promising route to application for the sequence perspective will require a shift in the thinking of the field that we are not yet seeing, similar to the shift from marker-assisted to genomic selection.

## Conclusions

In conclusion, there are (at least) two ways to think of genomics in animal breeding, that are helpful in understanding how genomic technologies have changed and may continue to change animal breeding. Currently, tools derived from the statistical perspective are doing the heavy lifting in breeding practice, in the form of genomic selection. With the advent of new technologies, the sequence perspective could make an impact in the future, if it can overcome the twin problems of how to identify causative variants for complex traits and how to introduce them into animals, both at scale.

## Data Availability

Not applicable.
