# Correction: Genomics in animal breeding from the perspectives of matrices and molecules

**DOI:** 10.1186/s41065-023-00287-8

**Published:** 2023-05-18

**Authors:** Martin Johnsson

**Affiliations:** Department of Animal Breeding and Genetics, Swedish University of Agricultural Sciences, Box 7023, Uppsala, 75007 Sweden

**Correction: Hereditas 160, 20 (2023).**

10.1186/s41065-023-00285-w.

Following publication of the original article [1], the author reported that the revised version with track changes of the manuscript was captured as an additional file. This should be removed.

The original article has been corrected.
